# Association of Maternal Sense of Coherence With Oral Health Behavior of Children With Special Health Care Needs: A Cross-Sectional Study

**DOI:** 10.7759/cureus.51635

**Published:** 2024-01-04

**Authors:** Kiran Iyer, Rana M Almutairi, Razan Eidah Alsaadi, Wafa Mubarak Alanazi, Afnan Saeed Alamri, Samar Muhammed Binzafer

**Affiliations:** 1 Dental Public Health, Preventive Dental Science Department, College of Dentistry, King Saud Bin Abdulaziz University for Health Sciences, King Abdullah International Medical Research Centre, Riyadh, SAU; 2 Dentistry, College of Dentistry, King Saud Bin Abdulaziz University for Health Sciences, Riyadh, SAU

**Keywords:** mothers, special health care needs children, arabic version, validation study, sense of coherence, saudi mothers

## Abstract

Background: Sense of coherence (SOC) has been adopted to analyze stress coping skills as well as to find its association with health and oral health behavior. The Arabic version of SOC has not been developed and adopted in the Saudi Arabian population; furthermore, few studies have attempted to analyze the association of a mother's sense of coherence with the oral health behavior of Special Care Health Needs (SCHN) children. Hence, this study aimed to observe the association of mothers' SOC scores with their children’s oral health behavior, along with the validation of the scale.

Materials and methods: A cross-sectional study was conducted among 170 mothers of children with SCHN. Participants were recruited from two schools in Riyadh City and one school in Makkah, respectively. Descriptive statistics, validation, reliability, factor analysis, and multinomial logistic regression were carried out using the Statistical Package for the Social Sciences (SPSS, Version 20, 2011; IBM Corp., Armonk, USA).

Results: The mean SOC-13 score was 61.6 (±10.1), with a median value of 61. The SOC scale elicited a Cronbach’s alpha value of 0.77. The Spearman-Brown-Brownuttman split-half reliability coefficients were found to be 0.70, respectively. The analysis confirmed a three-factor solution, which explains about 51.9% of the total variance. Mothers with higher SOC tend to utilize dental services for children [OR 5.69, P = 0.029, CI 1.19-27.02] and restrict sugary drinks to once a day [OR 9.31, P = 0.00, CI 1.95-44.44].

Conclusion: The reliability of the Arabic scale that was adopted after translation in the present study was found to be high; the scale confirms the three-factor solution. Mothers who scored low on the SOC scale tended to utilize less dental service for their children as well as overlook the sugary drink/day intake of the children.

## Introduction

As per the World Health Organization's Ottawa Charter for health promotion, the focus is to develop people's ability and opportunity to make healthy choices [1]. For children with Special Care Health Needs (SCHN), health-related choices are largely dependent on parents or caregivers. As per the definition, "special healthcare needs include any physical, developmental, mental, sensory, behavioral, cognitive, or emotional impairment or limiting condition that requires medical management, health care intervention, and/or use of specialized services or programs" [2]. Literature discloses poor oral health status and undesirable oral health behaviors to be common among children with SHCN [3].

Unfavorable oral health behavior has been associated with many factors, one of which is a lack of oral health knowledge. Maintenance of optimal oral health is also reliant on psychological and biopsychosocial models, such as health locus of control and sense of coherence (SOC). Antonovsky proposed the salutogenesis theory, which is a psycho-social approach that focuses on factors that support human health and well-being. SOC is pivotal to the salutogenic model [4].

The SOC consists of three components that estimate how individuals perceive the world (understanding), ascertain and use resources to meet the demand (management), and answer the questions emotionally (meaning) [5].

Studies from the past have shown an association between SOC and general health behavior [6,7]. Chronic diseases of the oral cavity are associated with non-biological risk factors [8]. Some studies have also established an association between SOC and oral health behavior (visits to the dentist, tooth brushing frequency), adolescent oral health behavior, and oral health-related quality of life. Maternal/caregiver SOC has been shown to influence the oral health behavior of children [1,3].

Studies have reported that adolescents whose mothers had higher SOC scores experienced fewer dental caries and gingivitis. Another study reported that mothers of children aged 11-12 with higher levels of SOC were more likely to use dental services. Studies have also shown an association between mothers' low SOC levels and tooth decay, pulpitis, or restored teeth in children [3,5,9].

Interestingly, few researchers have analyzed the strength of the association between maternal SOC and the oral health behavior of SCHN children. Assessing this association becomes important as these children are dependent on maternal care [10]. Hence, the aim was to observe if there is an association between maternal SOC and the oral health behavior of children with SCHN. As a part of the primary objective intended to address the aim, the study included the translation of the SOC-13 questionnaire into ARABIC and carried out its validation and reliability assessment, along with analyzing the construct of the scale.

## Materials and methods

A descriptive, cross-sectional, questionnaire-based study was conducted to assess the association between maternal SOC and oral health behavior in children with Special Health Care Needs. We obtained the requisite Institutional Review Board approval (IRB/1745/22) from King Abdullah International Medical Research Center (KAIMRC) for the study protocol before collecting data. Permission to approach the office of the special care center (elementary school, 381 for girls) was obtained through the Ministry of Education's (MOE) civil registry (Planning and Information Department, Letter:4401038320), and data were collected from two other schools (specialized day care center, Riyadh city, and middle school, 41, Makkah city) where participants were recruited for the study. Informed consent was obtained from mothers of children with SCHN who were willing to be enrolled; questionnaires were self-administered to these mothers.

Sampling, sample frame, and sample size

The data collection was done over three months with weekly reminders to the mothers through the school officials to mothers of children with SCHN recruited from two schools in Riyadh and one school in Makkah. The participants were recruited based on a non-probability, purposive sampling technique. Only those mothers with children who have special health care needs and who were willing to enroll in the study by providing informed consent were included in the study; caregivers other than the mothers and participants who returned incomplete questionnaires were excluded from the study. An estimated sample size of 170 was obtained based on correlation (r = 0.20) based on previous literature [11]. We estimated the sample size using G Power software (version 3.1.9.4; G Power, Düsseldorf, Germany).

Questionnaire

The SOC-13-item questionnaire [12] has been used in various countries and validated in their respective vernacular languages. This study attempted to translate the questionnaire into Arabic, as it has not been carried out in the past. A bilingual expert with a master's degree in linguistics (private translation center) helped translate the questionnaire into Arabic, and it was then back translated into English by another independent translator. Discussions were held between translators and investigators to arrive at a consensus. The back-translated questionnaire was compared with the original English version to check for the integrity of the questions during the translation process. Face, content, and construct validity were assessed. A total of 20 (11.4%) mothers (within the sample) were approached to assess test-retest reliability. The test-retest was conducted with a gap of four weeks, based on literature evidence. The internal consistency assessed through reliability was tested and reported using Cronbach’s alpha. Item-total statistics and item-delete statistics were calculated to check the correlation between the questions. A factor analysis was carried out to assess the response to the underlying philosophy.

Components and variables

The SOC-13 item questionnaire consists of three sections: demographic details, SOC-3 item scale, and a child's oral health-related behaviors. Demographic details included age, educational status, marital status, monthly family income, and nationality. Demographic variables were used as explanatory variables in the study. The SOC consists of three dimensions: comprehensibility (five items), manageability (four items), and meaningfulness (four items). All items were scored on a Likert scale ranging from 1 to 7. The sum of the scores for SOC varied from 13 to 91. A high score indicated a strong SOC. The SOC scale was the independent variable in the study. The final section of questions on children's oral health behavior consisted of the following five questions: one question on oral hygiene, two questions on diet, and two questions on dental attendance and utilization. Mothers do serve as a reliable source to seek out the oral health behavior of children, as substantiated by previous literature [13]. Response to questions on oral health behavior was the dependent variable in the study.

Statistical analysis

The data were entered in Microsoft Excel (Microsoft Corp., New York, USA) and transferred to Statistical Package for the Social Sciences (SPSS) statistical software (version 20, 2011; IBM Corp., Armonk, USA) for analysis. Descriptive statistics were used to describe the demographic data based on frequency and percentage. Validity testing, reliability assessment, and factor analysis were carried out for the scale after Arabic translation and adoption in the study. The maternal SOC score is a continuous variable; the total SOC was divided into two categories (low and high scores) and analyzed for significance with oral health behavior based on multinomial logistic regression analysis. As a second step, multinomial logistic regression was applied to assess the significance of maternal SOC scores with the exploratory variables.

## Results

Demographic details and components assessment

A total of 174 mothers responded to the self-reported questionnaire. After a three-month follow-up, the mean age of mothers who participated in the study was 37.3 (±6.2) years, and the mean age of their children was observed to be 8.0 (±3.7) years. The highest frequency of 90 (51.7%) for family income per month was observed to be the 5000-10,000 Saudi Arabian Riyal (SAR). Most of the respondents, 162 (93.1%), were Saudi-Arabian nationals; only wards of citizens can be enrolled in government schools; hence, it is safe to assume the remaining are citizens (though non-Saudi Arabian) as a part of the marital arrangement. Interestingly, a high frequency of mothers, 77 (44.3%), reported having completed their graduation when education qualification was analyzed. Similarly, a high number of respondents, 159 (91.4%), reported being married when the type of parenthood or motherhood was assessed among them (Table 1).

**Table 1:** Demographic variables in the study represented as mean, standard deviation, frequency and percentage.

Demographic variable		Total (N)	Mean	Standard deviation (±)	Frequency (n)	Percentage (%)
Age of mother		174	37.3	6.2	-	-
Age of child		174	8.0	3.7	-	-
Family income/month	<5000 SAR	174	-	-	33	19.0
	5000–10,000 SAR				90	51.7
	10,000–20,000 SAR				41	23.6
	>20,000 SAR				10	5.7
Type of motherhood	Single	174	-	-	15	8.6
	Married				159	91.4
Country of origin	Non-Saudi Arabia	174	-	-	12	6.9
	Saudi Arabia				162	93.1
Mothers education	Elementary school	174	-	-	16	9.2
	Secondary school				15	8.6
	High school				66	37.9
	Graduate				77	44.3

The mean SOC-13 score was found to be 61.6 (±10.1), with a median value of 61. When individual questions as against their components were assessed, two of the questions under meaningfulness (“Do you feel like you don't really care about what goes on around you?” and “Doing the thing you do every day is?”) had high mean scores of 5.3 (±1.2) and 5.3 (±1.5), respectively. Similarly high mean scores of 5.1 (±1.3) were observed for the question "Are your thoughts (ideas) and feelings very complex?" related to the comprehensibility component and the question "Do you have a feeling that you are not being treated fairly?" under the manageability component had the highest mean score of 4.9 (±1.4) (Table 2).

**Table 2:** Mean, median, and standard deviation for Items under components of SOC-13. SOC: sense of coherence.

Component (code)	SOC 13 - questions/items	Mean	Median	Standard deviation (±)
Meaningfulness (M1)	Do you feel like you don't really care about what goes on around you?	5.3	5.0	1.2
Comprehensibility (C1)	Where have you been surprised by the behavior of someone you know in the past?	4.2	4.0	1.3
Manageability (Mg 1)	Have people whom you trusted let you down (disappointed)?	4.3	4.0	1.6
Meaningfulness (M 2)	Until now, your life has had	4.9	5.0	1.5
Manageability (Mg 2)	Do you have the feeling that you are not being treated fairly?	4.9	5.0	1.4
Comprehensibility (C 2)	Do you have the feeling that you are in unfamiliar situation and don't know what to do?	4.1	4.0	1.4
Meaningfulness (M 3)	Doing the thing you do every day is?	5.3	6.0	1.5
Comprehensibility (C 3)	Are your thoughts (ideas) and feelings very complex?	5.1	5.0	1.3
Comprehensibility (C 4)	Have you ever felt that there are feelings inside you that you do not want to feel?	5.0	6.0	1.6
Manageability (Mg 3)	How have you felt like a (failure) disappointed in certain situation?	3.9	4.0	1.5
Comprehensibility (C 5)	When certain events happen, do you generally find that you are?	4.9	5.0	1.3
Meaningfulness (M 4)	How many times do you feel there is meaning to the things you do in your daily life?	4.8	5.0	1.5
Manageability (Mg 4)	How many times do you feel you weren’t sure you would be under control?	4.5	5.0	1.9
SOC-13 (total score)		61.6	61	10.1

The SOC questionnaire elicited a Cronbach’s alpha value of 0.77 for the reliability assessment of the 13 items. The Spearman-Brown and Guttman split-half reliability coefficients were found to be 0.70, respectively. The analysis of the test-retest on the sample revealed a reliability of 0.74 (P<0.00). The summary of inter-item correlations showed six negative correlations and the values ranged from minimum −0.168 to maximum 0.632. The lowest score of 80.3 (α=0.72) on scale variance under item-total correlations was for the question related to compressibility (“Are your thoughts (ideas) and feelings very complex?"); however, deleting any of the questions in the scale did not influence the highest overall observed Cronbach’s alpha value of 0.77 (Tables 3-4).

**Table 3:** Inter-item correlation matrix for items in SOC-13. SOC: sense of coherence; N: total.

Inter-item correlation matrix													
	Do you feel like you don't really care about what goes on around you?	Where have you been surprised by the behavior of someone you know in the past?	Have people whom you trusted let you down (disappointed)?	Until now, what has your life has had?	Do you have the feeling that you are not being treated fairly?	Do you have the feeling that you are in an unfamiliar situation and don't know what to do?	Doing the thing you do every day is?	Are your thoughts (ideas), and feelings very complex?	Have you ever felt that there are feelings inside you that you do not want to feel?	How have you felt like a (failure) disappointed in certain situation?	When certain events happen, do you generally find that you are?	How many times do you feel there is meaning to the things you do in your daily life?	How many times do you feel you weren’t sure you would be under control?
Do you feel like you don't really care about what goes on around you?	1.00	0.135	0.133	0.147	0.259	0.235	0.046	0.267	0.225	0.151	0.193	−0.168	0.213
Where you surprised by the behavior of someone you know in the past?	0.135	1.000	0.479	−0.118	0.213	0.116	−0.113	0.215	0.094	0.090	0.004	0.074	−0.052
Have people whom you trusted let you down (disappointed)?	0.133	0.479	1.000	0.101	0.456	0.156	0.129	0.202	0.145	0.064	0.070	0.050	0.031
Until now your life has had	0.147	−0.118	0.101	1.000	0.236	0.219	0.243	0.243	0.038	0.315	0.083	−0.066	0.166
Do you have feeling that you are not being treated fairly?	0.259	0.213	0.456	0.236	1.000	0.364	0.156	0.433	0.373	0.245	0.344	0.118	0.287
Do you have the feeling that you are in unfamiliar situation and don't know what to do?	0.235	0.116	0.156	0.219	0.364	1.000	0.188	0.575	0.632	0.394	0.327	0.041	0.395
Doing the thing you do every day is?	0.046	−0.113	0.129	0.243	0.156	0.188	1.000	0.280	0.182	0.270	0.125	−0.022	0.195
Are your thoughts (ideas) and feelings very complex?	0.267	0.215	0.202	0.243	0.433	0.575	0.280	1.000	0.459	0.495	0.426	0.204	0.470
Have you ever felt that there are feelings inside you that you do not want to feel?	0.225	0.094	0.145	0.038	0.373	0.632	0.182	0.459	1.000	0.231	0.350	0.091	0.219
How have you felt like a (failure) disappointed in certain situation?	0.151	0.090	0.064	0.315	0.245	0.394	0.270	0.495	0.231	1.000	0.262	0.044	0.470
When certain events happen, do you generally find that you are?	0.193	0.004	0.070	0.083	0.344	0.327	0.125	0.426	0.350	0.262	1.000	0.172	0.328
How many times do you feel there is meaning to the things you do in your daily life?	-0.168	0.074	0.050	−0.066	0.118	0.041	−0.022	0.204	0.091	0.044	0.172	1.000	0.287
How many times do you feel you weren’t sure you would be under control?	0.213	−0.052	0.031	0.166	0.287	0.395	0.195	0.470	0.219	0.470	0.328	0.287	1.000
Reliability statistics	Cronbach's Alpha	Cronbach's Alpha Based on Standardized Items	(N) of Items										
	0.770	0.772	13										

**Table 4:** Correlation as per the item - total statistics for the SOC-13 scale. SOC: sense of coherence.

Item-total statistics	Scale mean if the item deleted	Scale variance if item deleted	Corrected item-total correlation	Cronbach's alpha if the item deleted
Do you feel like you don't really care about what goes on around you?	56.32	94.520	0.289	0.764
Were you surprised by the behavior of someone you know in the past?	57.41	96.741	0.174	0.774
Have people whom you trusted let you down (disappointed)?	57.30	91.022	0.312	0.764
Until now your life has had	57.11	90.502	0.248	0.775
Do you have a feeling that you are not being treated fairly?	56.71	84.692	0.575	0.736
Do you have the feeling that you are in an unfamiliar situation and don't know what to do?	56.83	83.450	0.596	0.733
Doing the thing you do every day is?	56.67	93.819	0.270	0.767
Are your thoughts (ideas) and feelings very complex?	56.30	80.315	0.710	0.720
Have you ever felt that there are feelings inside you that you do not want to feel?	57.46	87.695	0.492	0.746
How have you felt like a (failure) disappointed in certain situations?	56.51	88.679	0.493	0.746
When certain events happen, do you generally find that you are?	56.59	86.786	0.427	0.752
How many times do you feel there is meaning to the things you do in your daily life?	57.68	96.821	0.127	0.782
How many times do you feel you weren’t sure you would be under control?	56.69	89.175	0.488	0.747

A highly convergent validity is said to be present for the given construct in the study, considering a Cronbach's alpha value of 0.77.

Factor analysis

**Figure 1:**
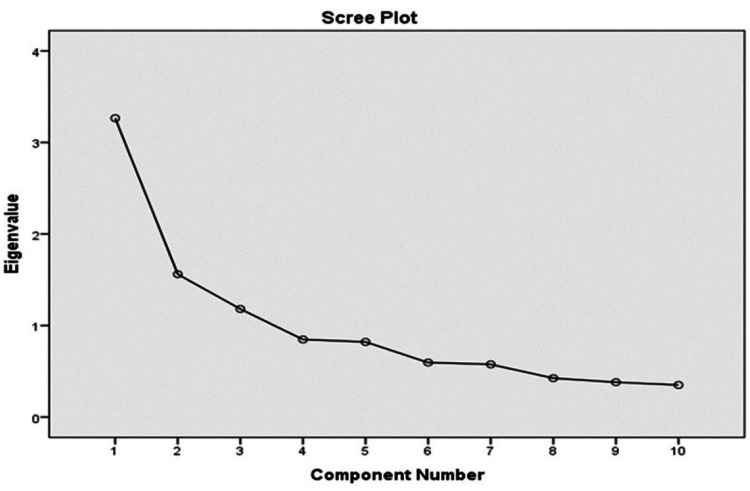
Scree plot indicating deviation from 3 factors, before adjusting for M1 and M3. M: meaningfulness.

The data satisfied the initial fitness for factor analysis, which was confirmed by a KMO sampling adequacy value of 0.74, Bartlett’s test of sphericity (chi-square −599.73), and P = 0.000. The analysis confirmed a three-factor solution, which explains about 51.9% of the total variance in the questionnaire scale. The extraction was carried out based on fixed factors. Factor 1 (meaningfulness) contributed to about 25.06% of the variance, whereas factor 2 and factor 3 contributed to 14.49% and 12.4% of the total variance, respectively. Factor analysis was also carried out using eigenvalues >1, which resulted in four four-factor solutions. This was mainly seen due to the non-contribution of M1 and M3 as observed in the rotation matrix as well as the principal component analysis (M1 = 0.319 and M3 = 0.326). The scree plot is indicative of a three-factor solution when adjusted for (M1, M3, C4, and C5) and just about corroborates when these four questions are added; a one-factor solution as initially indicated by Antonovsky for SOC was unsatisfactory. Factor loadings do suggest some of the questions do not correspond with the underlying theoretical basis of the three factors (Tables 5-7) (Figure 1).

**Table 5:** Total variance explained for each of the SOC-13 questions by factor analysis. SOC: sense of coherence.

Component	Initial eigenvalues			Rotation sums of squared loadings			
	Total	% of variance	Cumulative %	Total	% of variance	Cumulative %	
1	3.850	29.618	29.618	3.259	25.069	25.069	
2	1.603	12.331	41.950	1.884	14.493	39.562	
3	1.305	10.041	51.991	1.616	12.429	51.991	
4	1.113	8.565	60.556				
5	0.921	7.086	67.642				
6	0.839	6.451	74.093				
7	0.736	5.662	79.756				
8	0.651	5.009	84.765				
9	0.556	4.278	89.043				
10	0.428	3.289	92.332				
11	0.395	3.040	95.371				
12	0.326	2.507	97.879				
13	0.276	2.121	100.000				

**Table 6:** Principal component analysis for SOC-13 scale in the study. Extraction method: principal component analysis. *Items contributing to deviation from 3 factors. SOC: sense of coherence.

Communalities	Initial	Extraction
M 1	1.000	0.319*
M 2	1.000	0.486
M 3	1.000	0.326*
M 4	1.000	0.630
C 1	1.000	0.667
C 2	1.000	0.564
C 3	1.000	0.675
C 4	1.000	0.433
C 5	1.000	0.412
Mg 1	1.000	0.663
Mg 2	1.000	0.542
Mg 3	1.000	0.479
Mg 4	1.000	0.561

**Table 7:** Rotated component matrix based on Varimax rotation for SOC-13 scale. Extraction method: principal component analysis. Rotation method: varimax with Kaiser normalization. Rotation converged in 5 iterations. SOC: sense of coherence.

	Component		
	1	2	3
C 3	0.758		
C 1	0.792		
C 2	0.670		
C 5	0.640		
C 4	0.597		
Mg 3	0.578		
Mg 1		0.809	
Mg 4		0.735	
Mg 2		0.539	
M 2			0.674
M 4			−0.621
M 3			-
M 1			-

A multinomial logistic regression analysis was carried out to analyze the SOC scores against the exploratory variables. Initially, the SOC scores were dichotomized into low (13-46) and high (47-91); dichotomization for analysis has been carried out in the past for SOC scores [1]. Exploratory variables analyzed were the type of motherhood (P>0.05), mothers’ educational status (P>0.05), and monthly family income (P=0.003). It was observed that those with incomes of less than 5000 SAR [OR 1.52, P=0.00, CI 4.57-5.06] and 5000-10,000 SAR [OR 1.48, P=0.00, CI 2.94-7.52] are more likely to have scored lower on the SOC scale (Table 8). In the present study, interestingly, the mother's education was not a confounder of the children's oral health behavior, whereas, on initial assessment by chi-square analysis, monthly family income had a significant relationship with the child's sugar drink consumption frequency and brushing habit. However, when adjusted in the regression analysis, sweet drinks were significantly [OR 3.02, P=0.00, CI 2.33-18.29] less likely to be consumed by children from higher-income households.

**Table 8:** Multinomial regression to assess significance of mother’s SOC scores with exploratory variables and child’s oral health behavior. The first category was considered the reference group with a low SOC score of 13–46. *P<0.05 is significant (Sig). Exp (B): odds; df: degrees of freedom; B: regression coefficients.

Variable		B	df	Sig.	Exp (B)	95% confidence interval for Exp (B)	
						Lower bound	Upper bound
Family income/month	<5000 SAR	−18.00	1	0.00*	1.52	4.57	5.06
	5000–10,000 SAR	−15.72	1	0.00*	1.48	2.94	7.52
	10000–20,000 SAR	−17.92	1	0.69	1.64	0.94	1.54
	>20,000 SAR	0	0	-	-	-	-
How often does your child have sweet drinks/juice?	Once a day	2.23	1	0.00*	9.31	1.95	44.41
	More than once a day	0.58	1	0.48	1.79	0.34	9.31
	Don’t drink	0	0	-	-	-	-
Do you utilize dental services for children?	Yes	1.73	1	0.02	5.69	1.19	27.02
	No	0	0	-	-	-	-
Type of motherhood	Single mother	−0.66	1	0.48	0.51	0.80	3.29
	Married	0	0	-	-	-	-
Mother’s educational status	Elementary school	−0.10	1	0.91	0.90	0.12	6.37
	Secondary school	0.03	1	0.97	1.03	0.15	6.92
	High school	0.27	1	0.70	1.31	0.32	5.35
	Graduate	0	0	-	-	-	-

Similarly, SOC scores were analyzed against children’s oral health behavior as reported by mothers. Two questions elicited significant responses: those who scored higher on the SOC scale were more likely to use dental services than those who scored lower: “Do you utilize dental services for children?” [OR 5.69, P=0.029, CI 1.19-27.02] and “Does your child have sweet drinks?” [OR 9.31, P=0.00, CI 1.95-44.44] (Table 8).

## Discussion

The study attempted to satisfactorily translate the SOC scale into Arabic when this research was proposed for ethical approval. There were known studies done on this topic in this region; subsequently, two studies have come to light: one Arabic SOC-13 scale validated and studied in the United Arab Emirates (UAE) population [14], and subsequently, the same scale was used for validation in this region (Saudi Arabia) [15]. However, there are no reported studies that have developed (translated), applied, and validated the scale specific to this population. Social factors are known to influence salutogenesis and, in turn, reflect on the response to the SOC scale [8].

At the core of Antonovsky’s salutogenesis model lies a sense of coherence; it assesses the stressors present in people’s lives and how they cope with them to remain healthy. The same has been adopted to assess oral health behaviors and practices in the past [1]. The SOC has been adopted in 33 different languages across 32 countries [16]. The SOC-29 and SOC-13 item scales are the most studied. This study adopted the SOC-13 scale not only for convenience but also since the scale has been reliable when tested in various populations (α = 0.70 to 0.92) [16]. Our study did observe a good reliability score (Cronbach’s α value = 0.77), which is consistent with most studies as well as with the two other studies done in the region [14,15].

Also, the present study attempted to analyze the underlying psychometric properties of the translated scale through factor analysis, along with using the scale to assess the mothers' SOC and its association with their children's oral health behavior. One study in the region, as aforementioned, attempted the factor analysis by adopting an Arabic SOC-13 scale developed and validated among the UAE population. Psychometric analysis testing among various populations would add further to the reliability of the scale [17].

When inter-item correlation was assessed, all correlations were found to be positive except for three negative correlations (M1-M4, C1-M2, and C1-M3). This could explain the poor performance of M1 and M3 in eliciting responses and not contributing towards a three-factor solution. The present study's inter-item correlation findings are in line with a study by Naaldenberg et al. [18]. However, certain studies, such as Rajesh et al. [19] and Tang and Dixon et al. [20], have reported a higher inter-item correlation for the scale when subjected to their respective populations. The first item under meaningfulness (M1) has consistently been reported to have a poor correlation with the whole construct, as reported in studies by Alharbi et al. [15] and Lerdal et al. [21]. This finding is in line with our observations in the study. The SOC-12 item scale was developed by dropping the first item (M1) and has been used in many studies to assess the sense of coherence and its association with health [1,22].

However, deleting any of the items did not contribute to a higher Cronbach’s alpha value than the overall reliability value of 0.77 observed. Factor analysis initially revealed a four-factor solution at eigenvalues >1. Mahammadzadeh and Poursharifi [23] also reported a similar observation. One Swedish study also reported a five-factor solution [24]. A fixed-factor analysis was carried out in the study to explain the variance in a three-factor solution. Through the principal component analysis, a 51.9% variance could be explained by the three-factor solution in our study. This observation is also in line with a study by Mahammadzadeh et al. [23]. Most studies have reported concurrence with the three-factor solution as well as lower and higher variance, respectively [1,15,19]. Most studies raise concern for variance in correlation with certain questions in the scale [21,23]. A one-factor solution has also been proposed by Eriksson and Lindström [16], but no studies have fully found a concurrence with the same; this too was analyzed in the present study and failed to confirm the same. The observations of the one-factor solution observed in our study are in line with other studies [15,19].

The study demonstrated that a mother's sense of coherence has a significant association with certain oral health behaviors of their children. Mothers with higher levels of sense of coherence significantly reported providing sugary drinks/juice once a day and were significantly more likely to utilize dental services for their children than those who scored lower on the scale. These findings of oral health behavior observed in our study are in line with a study by Lindmark et al. [1], who observed lower sugary drinks consumed by those who scored higher on the SOC scale, as well as significantly higher utilization of dental services with less anxiety in this group. A study by Qiu et al. [3] contrastingly reported no significance between mothers' SOC and sugary snack intake. Our observation of the utilization of dental services by mothers with higher SOC is in line with studies by Da Silva et al. [25] and Freire et al. [26]. Yet another notable oral health behavior of snacking between meals was significantly lower in the group with a higher SOC, as reported by Lindmark et al. [1]. This variable was assessed in the present study but had no significance for mothers' SOC.

Mothers' sense of coherence has been shown to have a bearing on the oral health behavior and quality of life of the children. Even though the limitation of such studies is that they are self-reported, it is still a reliable way to analyze, as assessed and recommended by previous studies [11,13,27].

Limitations

Antonovsky originally never intended or recommended that the SOC scale be examined by dividing the values into low or high. However, studies have divided the scores broadly in two ways, such as low, intermediate, and high, as well as low and high, to establish associations. This drawback leads to inconsistency in drawing associations and making comparisons between the findings of various populations. This is a cross-sectional study, which is a limitation in itself. The sampling procedure employed in this study was purposeful, as we required mothers of children with SCHN. Future studies can undertake cluster-based samples and stratify based on disability to see the variation in SOC scores and their association with oral health behavior. Two of the meaningfulness subscale questions (M1 and M3) were laggards and needed to be addressed with input from Arabic experts to make them more understandable and easier to respond to, which could further enhance the reliability of the scale. This scale can be validated on larger samples for confirmation and to further strengthen the observations from the present study.

## Conclusions

This study adds strength to SOC psychometric analysis. The reliability of the Arabic scale that was adopted after translation in the present study was found to be high; the scale confirms a three-factor solution and shows an inclination towards a four-factor solution as a couple of questions from the meaningfulness subscale are not contributing much towards the convergence. Mothers overall had a high median SOC score, and it was observed that those who scored low on the SOC scale tended to utilize less dental service for their children as well as overlook the sugary drink/day intake of the children. Factors such as the brushing habits of the children, consumption of biscuits, and between-meal snacking were not significant in the present study.
